# Musculoskeletal pain intensity in different body regions and risk of disability pension among female eldercare workers: prospective cohort study with 11-year register follow-up

**DOI:** 10.1186/s12891-021-04655-1

**Published:** 2021-09-10

**Authors:** Rúni Bláfoss, Jonas Vinstrup, Sebastian Venge Skovlund, Rubén López-Bueno, Joaquin Calatayud, Thomas Clausen, Lars Louis Andersen

**Affiliations:** 1 National Research Centre for the Working Environment, Lersø Parkallé 105, 2100 Copenhagen, Denmark; 2 Research Unit for Muscle Physiology and Biomechanics, Department of Sports Science and Clinical Biomechanics, University of Southern Denmark, Odense, Denmark; 3 Department of Physical Medicine and Nursing, University of Zaragoza, Zaragoza, Spain; 4 Exercise Intervention for Health Research Group (EXINH-RG), Department of Physiotherapy, University of Valencia, Valencia, Spain; 5 Sport Sciences, Department of Health Science and Technology, Aalborg University, Aalborg, Denmark

**Keywords:** Musculoskeletal diseases, Health personnel, Working life, Work ability, Pensions

## Abstract

**Background:**

Musculoskeletal pain is a risk factor for leaving the labour market temporarily and permanently. While the presence of multi-site pain increases the risk of disability pension, we lack detailed knowledge about pain *intensity* as a risk factor. This study investigated the association between musculoskeletal pain intensity in different body regions and risk of future disability pension among eldercare workers.

**Methods:**

Eight thousand seven hundred thirty-one female eldercare workers replied to a questionnaire on work and health in 2005 and were followed for 11 years in the Danish Register for Evaluation of Marginalization. Time-to-event analyses estimated hazard ratios (HR) for disability pension from pain intensities (0–9 numeric rating scale (NRS)) in the low-back, neck/shoulders, and knees during the previous 3 months. Analyses were mutually adjusted for pain regions, age, education, lifestyle, psychosocial work factors, and physical exertion at work.

**Results:**

During 11-year follow-up, 1035 (11.9%) of the eldercare workers received disability pension. For all body regions among all eldercare workers, dose-response associations were observed between higher pain intensity and risk of disability pension (*p* < 0.001). The risk for disability pension was increased when reporting “very high” pain levels (≥7 points on the 0–9 NRS) in the low-back (HR 2.19, 95% CI 1.70–2.82), neck/shoulders (HR 2.34, 95% CI 1.88–2.92), and knees (HR 1.89, 95% CI 1.44–2.47). Population attributable risks (PAR) were 15.5, 23.2, and 9.6% for pain > 2 on NRS in the low-back, neck/shoulders, and knees, respectively, indicating that 15.5, 23.2, and 9.6% fewer eldercare workers would likely receive disability pension if the pain intensity was reduced to 2 or less. For workers ≤45 years and > 45 years, PAR was highest for neck/shoulder pain (27.6%) and low-back pain (18.8%), respectively.

**Conclusions:**

The present study found positive dose-response associations between pain intensity in the low-back, neck/shoulders, and knees, and risk of disability pension during 11-year follow-up. Moderate to very high levels of musculoskeletal pain in eldercare workers should, therefore, be considered an early warning sign of involuntary premature exit from the labour market. These findings underscore the importance of preventing, managing, and reducing musculoskeletal pain to ensure a long and healthy working life.

## Background

The gradually increasing statutory pension age in most European countries increases the necessity of staff retention and thus maintenance of good health of an aging workforce. However, musculoskeletal pain is common in the working population and the prevalence of pain increases with age [1]. More specifically, workers with physically demanding occupations are at a particularly high risk of both musculoskeletal pain [2, 3], long-term sickness absence, and disability pension compared to workers within more sedentary occupations [4–6]. Granted that both age and physically demanding work are predictors of musculoskeletal pain, these factors – especially in combination – constitute significant risk factors for being unable to work until statutory pension age. In fact, musculoskeletal pain located in different body regions, such as low-back, neck/shoulders, and knees has been associated with reduced work ability in a dose-response fashion [7, 8] and elevated risk of sickness absence [9, 10], both of which being associated with an increased risk of disability pension [11–14]. In accordance, two recent studies found musculoskeletal pain to be associated with an increased risk of disability pension [15, 16], one of which reporting a dose-response association between *number* of pain regions and increased risk of disability pension [15]. Thus, workplaces should be aware of workers in pain and aim to prevent and reduce the incidence of musculoskeletal pain among the workers.

Certain job groups report particularly high physical work demands and prevalence of musculoskeletal disorders. One of these job groups are healthcare workers who are at increased risk of musculoskeletal pain [17–19], sickness absence [9, 20], and disability pension [21–23]. In the latest (2018) round of the Work Environment and Health study in Denmark, 46% of healthcare workers reported having musculoskeletal pain several times per week, consequently representing the job group with the highest prevalence of weekly bodily pain [24]. In addition, 9% of the healthcare workers felt limited during work due to pain [24]. The high prevalence of musculoskeletal pain among healthcare workers may represent an even greater challenge in the future due to the demographic changes in most European countries with an increased proportion of older residents and thereby increased need for eldercare, potentially resulting in societies becoming short of healthcare workers [25].

The high prevalence of pain among healthcare workers may be explained by the high mental and physical demands they are confronted with in their daily work, comprising lifting and manually assisting persons – oftentimes with sustained and awkward postures [17, 18]. For instance, high physical exertion during eldercare work increased the odds of persistent low-back and knee pain (> 30 days during the past year) at 1-year follow-up [18], while heavy physical work and strenuous work postures increased the risk of disability pension among healthcare workers (including nurses) [21–23]. Additionally, musculoskeletal pain may also origin from chronic diseases such as osteoarthritis and cancer [26, 27]. The prevalence of sickness absence among nurses’ aides gradually increased during the 104 weeks before start of disability pension in a 15-year register follow-up cohort study [23]. These findings indicate that healthcare work is not only costly for the society and workplaces due to an increased risk of disability pension, but also due to a gradually increasing prevalence of sickness absence for at least 2 years before ending on disability pension [23]. This underlines the importance of addressing risk factors in the working environment that may predispose healthcare workers for ill health and hence push them out of the labour market prematurely. Since risk factors varies across job groups and the debilitating effect of the risk factors depends on the physical demands during work [28, 29], investigating risk factors in specific job groups provide valuable job-specific knowledge that can help to target suitable initiatives to reduce debilitating risk factors. Furthermore, both longer duration of pain [20] and pain intensity [9] in different body regions can have different consequences in relation to sickness absence among healthcare workers in eldercare (from now on called “eldercare workers”). In addition, work ability (proxy for disability pension) among nurses declined with higher *intensity* of musculoskeletal pain [30], while pain *intensity* in the low-back, neck/shoulders, and legs associated with increased work limitation (also a proxy for disability pension) in a dose-response manner among workers ≥50 years from the general working population [28, 29, 31]. Thus, while pain intensity is associated with poorer ability to work [28–31], and physical work demands and musculoskeletal pain (single- and multi-site pain) increase the risk of disability pension [15, 16, 21–23], no studies to date have investigated the dose-response association between pain *intensity* in different body regions and risk of disability pension among eldercare workers.

Thus, the aim of the present study was to investigate whether a dose-response association exists between pain intensity in the low-back, neck/shoulders, and knees, and disability pension among Danish eldercare workers. We hypothesized that higher pain intensity in all body regions is associated with higher risk of disability pension in a dose-response manner.

## Methods

### Study design and population

The present study is a prospective cohort study among eldercare workers with 11-year register follow-up using the Danish Register for Evaluation of Marginalization (DREAM); a national register on social transfer payments [32]. Questionnaires were sent to 12,744 eldercare workers in Denmark between late 2004 and spring 2005, where 9949 (78%) responded the questionnaire [22]. The present study excluded all male workers (*N* = 234) and workers who did not directly engage in care services (*N* = 1021, some of these were males). Thus, this study included 8731 (69%) female eldercare workers. Eldercare workers comprised social and healthcare assistants and helpers, other care helpers with no or short-term education, and nurses or therapists [22]. Because all participants did not reply to all questions, the number of participants for each analysis varies. The reporting of the study follows the STROBE recommendations [33].

### Ethics approval and consent to participate

According to the Danish legislation, research questionnaire- and register-based studies do not need approval from neither ethical and scientific committees nor informed consent (https://www.nvk.dk/~/media/NVK/Dokumenter/Vejledning_Engelsk.pdf) [34]. The study was notified to and registered at The Danish Data Protection Agency. All data were de-identified and analysed anonymously.

### Risk factors

#### Musculoskeletal pain

Participants rated their average musculoskeletal pain intensity during the last 3 months in the low-back, neck/shoulders, and knees on a 0–9 numeric rating scale (NRS), where 0 indicated ‘no pain’ and 9 indicated ‘worst imaginable pain’ [9]. The scale was horizontally oriented to illustrate a modified visual-analogue scale [9, 35]. In the questionnaire, the three body regions were illustrated on a drawing from the Nordic Questionnaire [36]. We collapsed the pain intensities into the intervals 0–2 (‘No or low pain’), 3–4 (‘Moderate pain’), 5–6 (‘High pain’) and 7–9 (‘Very high pain’) in the subsequent analyses.

### Outcome variable

#### Disability pension

Disability pension rates were obtained from the DREAM register, which contains weekly information on granted disability benefits, sickness absence, employment, education etc. for Danish residents. The DREAM register and the inclusion requirements were recently described in detail [22]. In the present study, we defined ‘disability pension’ as receiving any form of disability benefits which require full or partial loss of work ability, i.e. sheltered jobs, flex jobs and variants hereof, and full disability pension comprising 13 categories of disability benefits payment in DREAM [22].

### Confounders

The analyses were controlled for relevant confounders from the baseline questionnaire including age (years, continuous variable), body mass index (BMI) (kg/m^2^, continuous variable), smoking (dichotomous variable: ‘Yes’/‘No’), education (categorical variables: e.g. social and healthcare assistant, nurse, therapist, none), leisure-time physical activity (categorical: low, moderate, and high level), pain intensity in other body regions (numeric rating scale: 0–9), psychosocial work factors (continuous variable (0–100): emotional demands, influence at work, role conflicts, quality of leadership) from the Copenhagen Psychosocial Questionnaire [37], and physical exertion at work (Hollmann Index scale 0–62, where 62 is highest exertion and 0 is lowest) [9, 15, 22].

### Statistical analyses

We used the Cox proportional hazards model to estimate the hazard ratios (HR) and 95% confidence intervals (95% CI) of the pain intensity variables for receiving disability pension during follow-up. The time unit used in the Cox models is weeks, as the register provides week-to-week information throughout the follow-up period. The follow-up was 11 years or until censoring, which comprises death, voluntary early retirement pension, state pension, or emigration. Potential registered disability benefit payment within the follow-up period were non-censored and referred to as event times [22]. Organizational unit to which each worker belonged was added as a clustering factor using the *random* statement. Estimation method was maximum likelihood and the PHREG procedure of SAS 9.4 (SAS Institute, Cary, NC, USA) was used. Model 1 was adjusted for age and pain intensity of the three pain regions (mutually adjusted). Model 2 adjusted for the same factors as model 1 and additionally for education, smoking, BMI, leisure-time physical activity, psychosocial work factors (emotional demands, influence at work, role conflicts, quality of leadership), and physical exertion at work. We performed a trend test to investigate whether a dose-response association existed between pain intensity in different body regions and risk of disability pension. Additionally, we performed stratified analyses for eldercare workers ≤45 years and > 45 years. An alpha level of < 0.05 was considered statistically significant. Population attributable risks (PAR) were calculated based on the HRs and proportion exposed (P_e_) from model 2: PAR = P_e_(HR_e_-1)/(1 + P_e_(HR_e_-1)). P_e_ included all the numbers of pain intensity above 2.

## Results

Participant characteristics are reported in Table 1. Mean age at baseline was 45.4 years, and 11.9% of the female eldercare workers received disability pension during the 11-year follow-up. The prevalence of moderate or more intense musculoskeletal pain in the low-back, neck/shoulders, and knees was 50.4, 54.0, and 23.9%, respectively (Table 2).

**Table 1:** Participants, lifestyle- and work characteristics

	N	Mean	SD	%
Age	8731	45.4	10.0	
**Lifestyle factors**				
Smoking	8634			
*Yes*	3188			36.9
*No*	5446			63.1
BMI	8334	24.9	4.4	
Physical activity during leisure	8564			
*Low-intensity*	4020			46.9
*Moderate-intensity*	4149			48.5
*High-intensity*	395			4.6
**Musculoskeletal pain (0–9)**				
Low-back pain	8524	2.6	2.3	
Neck/shoulder pain	8577	2.9	2.5	
Knee ﻿pain	8558	1.3	2.1	
**Work factors**				
Physical exertion during work [1–7]	8376	3.9	1.2	
Psychosocial work factors (0–100)				
*Emotional demands*	8629	50.0	18.5	
*Influence at work*	8630	45.0	20.6	
*Role conflicts*	8647	41.5	15.7	
*Quality of leadership*	8444	56.9	21.8	
Disability pension	1035			11.9

*BMI* Body mass index

*SD* Standard deviation

**Table 2:** Association between pain intensity in low-back, neck/shoulders, and knees, and risk of disability pension for all eldercare workers

Body region	Pain intensity	N	%	Model 1		Model 2		PAR
				HR	95% CI	HR	95% CI	
Low-back	No or low pain (0-2)	4231	49.6	1		1		15.5%
	Moderate pain (3-4)	2411	28.3	1.17	(0.99–1.38)	**1.20**	**(1.00–1.44)**	
	High pain (5-6)	1383	16.2	**1.35**	**(1.12–1.63)**	**1.35**	**(1.10–1.67)**	
	Very high pain (7-9)	499	5.9	**2.23**	**(1.77–2.80)**	**2.19**	**(1.70–2.82)**	
Neck/shoulders	No or low pain (0-2)	3944	46.0	1		1		23.2%
	Moderate pain (3-4)	2197	25.6	1.10	(0.92–1.31)	**1.23**	**(1.01–1.50)**	
	High pain (5-6)	1598	18.6	**1.45**	**(1.21–1.75)**	**1.60**	**(1.31–1.96)**	
	Very high pain (7-9)	838	9.8	**2.20**	**(1.80–2.68)**	**2.34**	**(1.88–2.92)**	
Knees	No or low pain (0-2)	6516	76.1	1		1		9.6%
	Moderate pain (3-4)	1145	13.4	**1.34**	**(1.12–1.59)**	**1.26**	**(1.04–1.53)**	
	High pain (5-6)	578	6.8	**1.57**	**(1.27–1.95)**	**1.56**	**(1.24–1.97)**	
	Very high pain (7-9)	319	3.7	**1.92**	**(1.49–2.46)**	**1.89**	**(1.44–2.47)**	

Model 1: Adjusted for age and each of the three pain regions

Model 2: Model 1 + education + smoking + BMI + leisure time physical activity + psychosocial work factors (emotional demands, influence at work, role conflicts, quality of leadership) + physical exertion at work

*HR* Hazard ratio

*PAR* Population attributable risk

Statistically significant HRs are marked with **bold**

The baseline pain intensities as predictors for disability pension during the 11-year follow-up are provided in Table 2. Irrespective of pain region, a positive dose-response association was found between pain intensity and disability pension (trend test *p* < 0.001). For example, the fully adjusted model showed increased risk of disability pension, with HRs of 1.23 (95% CI 1.01–1.50), 1.60 (95% CI 1.31–1.96), and 2.34 (95% CI 1.88–2.92) for moderate, high, and very high pain intensity in the neck/shoulders, respectively. Furthermore, PARs for low-back, neck/shoulders, and knees were 15.5, 23.2, and 9.6%, respectively. Thus, if pain intensity in eldercare workers could be reduced to 2/9 or less, potentially 15.5, 23.2, and 9.6% fewer eldercare workers would have to receive disability pension.

Tables 3 and 4 provide pain intensities as predictors for future disability pension when stratified by age (≤45 years and > 45 years). The HRs and PARs for the eldercare workers ≤45 years were highest when having pain in neck/shoulders. PAR for pain in low-back, neck/shoulders, and knees among workers ≤45 years were 10.9, 27.6, and 9.3%, respectively (Table 3). For eldercare workers > 45 years, high and very high pain in all three body regions were predictors for future disability pension, while PAR was highest for low-back pain. PAR for pain in low-back, neck/shoulders, and knees were 19.7, 18.8, and 9.9%, respectively.

**Table 3:** Association between pain intensity in low-back, neck/shoulders, and knees, and risk of disability pension for eldercare workers ≤45 years

				Model 1		Model 2		
Body region	Pain intensity	N	%	HR	95% CI	HR	95% CI	PAR
Low-back	No or low pain (0-2)	1986	49.8	1		1		10.9%
	Moderate pain (3-4)	1166	29.3	1.11	(0.85–1.45)	1.15	(0.86–1.53)	
	High pain (5-6)	627	15.7	1.31	(0.97–1.77)	1.26	(0.90–1.77)	
	Very high pain (7-9)	208	5.2	**1.84**	**(1.23–2.74)**	**1.73**	**(1.12–2.70)**	
Neck/shoulders	No or low pain (0-2)	1856	46.4	1		1		27.6%
	Moderate pain (3-4)	1059	26.5	1.12	(0.84–1.50)	1.24	(0.90–1.72)	
	High pain (5-6)	708	17.7	**1.72**	**(1.28–2.30)**	**1.92**	**(1.40–2.65)**	
	Very high pain (7-9)	374	9.4	**2.27**	**(1.63–3.16)**	**2.66**	**(1.85–3.82)**	
Knees	No or low pain (0-2)	3265	81.9	1		1		9.3%
	Moderate pain (3-4)	431	10.8	**1.36**	**(1.00–1.86)**	1.24	(0.88–1.75)	
	High pain (5-6)	198	5.0	**1.70**	**(1.16–2.51)**	**1.92**	**(1.28–2.87)**	
	Very high pain (7-9)	93	2.3	**2.15**	**(1.34–3.47)**	**2.33**	**(1.40–3.87)**	

Model 1: Adjusted for age and each of the three pain regions

Model 2: Model 1 + education + smoking + BMI + leisure time physical activity + psychosocial work factors (emotional demands, influence at work, role conflicts, quality of leadership) + physical exertion at work

*HR* Hazard ratio

*PAR* population attributable risk

Statistically significant HRs are marked with **bold**

**Table 4:** Association between pain intensity in low-back, neck/shoulders, and knees, and risk of disability pension for eldercare workers > 45 years

Body region	Pain intensity	N	%	Model 1		95% CI	Model 2	PAR
				HR		HR	95% CI	
Low-back	No or low pain (0-2)	2245	49.5	1	(0.98–1.51)	1		19.7%
	Moderate pain (3-4)	1245	27.4	1.22	**(1.09–1.77)**	1.25	(0.98–1.58)	
	High pain (5-6)	756	16.7	**1.39**	**(1.85–3.29)**	**1.46**	**(1.12–1.90)**	
	Very high pain (7-9)	291	6.4	**2.47**		**2.57**	**(1.87–3.54)**	
Neck/shoulders	No or low pain (0-2)	2088	45.6	1	(0.84–1.32)	1		18.8%
	Moderate pain (3-4)	1138	24.9	1.05	**(1.01–1.62)**	1.18	(0.92–1.51)	
	High pain (5-6)	890	19.4	**1.28**	**(1.60–2.66)**	**1.37**	**(1.06–1.78)**	
	Very high pain (7-9)	464	10.1	**2.07**		**2.13**	**(1.61–2.83)**	
Knees	No or low pain (0-2)	3251	71.1	1	**(1.05–1.61)**	1		9.9%
	Moderate pain (3-4)	714	15.6	**1.30**	**(1.16–1.95)**	1.25	(0.98–1.58)	
	High pain (5-6)	380	8.3	**1.50**	**(1.36–2.46)**	**1.41**	**(1.06–1.88)**	
	Very high pain (7-9)	226	4.9	**1.83**	**(1.36–2.46)**	**1.74**	**(1.26–2.41)**	

Model 1: Adjusted for age and each of the three pain regions

Model 2: Model 1 + education + smoking + BMI + leisure time physical activity + psychosocial work factors (emotional demands, influence at work, role conflicts, quality of leadership) + physical exertion at work

*HR* Hazard ratio

*PAR* population attributable risk

Statistically significant HRs are marked with **bold**

## Discussion

The present study investigated musculoskeletal pain *intensity* as a risk factor for disability pension among female eldercare workers. To our best knowledge, this is the first study to demonstrate a dose-response association between pain intensity in low-back, neck/shoulders, and knees, and future risk of disability pension among eldercare workers. Furthermore, the PAR analyses indicated that 15.5, 23.2, and 9.6% of disability pensions could be spared if pain intensities were reduced to ‘No or low pain’ (0–2 on 0–9 scale) in the low-back, neck/shoulders, and knees, respectively. When stratified by age, PAR was highest for pain in neck/shoulders among workers ≤45 years (27.6%), while PAR was highest for pain in low-back among workers > 45 years (19.7%).

The highly significant dose-response association (*p* < 0.001) and the high PAR documented in this study confirm the large consequences of musculoskeletal pain among eldercare workers. Musculoskeletal pain was not only highly prevalent at baseline (i.e. 50.4, 54.0, and 23.9% having at least moderate pain in low-back, neck/shoulders, and knees, respectively), it was also associated with a more than two-fold increased risk of leaving the labour market prematurely within 11 years of follow-up. Having moderate pain intensity or higher in the neck/shoulders and knees significantly increased the risk of disability pension among all eldercare workers, whereas a significantly increased risk of disability pension was only evident in individuals reporting high (5–6/9) or very high (7–9/9) low-back pain. These findings are somewhat in agreement with a previous study that found a higher pain intensity threshold for the low-back (5/9) associated with an increased risk of long-term sickness absence than the corresponding thresholds for pain in neck/shoulders and knees (4 and 3, respectively) at 1-year follow-up among the same group of workers as the present study [9]. These findings could indicate that moderate pain in the low-back is more tolerable and less debilitating among eldercare workers than pain in neck/shoulders and knees, which may be explained by differences in physical work demands between the investigated muscle groups. Thus, low-back muscles may serve a more postural role during healthcare work while neck/shoulders and knees have a more active role during the work tasks, e.g. during lifting/assisting and walking. Another rationale may be that many suffer from non-specific low-back pain that subsides and recurs during their lifetime [38] whereas pain in neck/shoulders and knees to a larger extent may originate from osteoarthritis or other degenerative chronic musculoskeletal disorders, which tend to progress negatively with age [26]. Contrastingly, our age-stratified analyses show highest PAR for pain in low-back in the older workers (> 45 years) and highest PAR for neck/shoulder pain in the younger workers (≤45 years). However, the PAR for pain in neck/shoulders is approximately at the same level for the older workers, whereas PAR for low-back pain and knee pain are markedly higher among the older eldercare workers compared to their younger counterparts. This corresponds with recent findings that pain progresses with age [26] and that high physical exertions have worse consequences for older workers [39]. Another argument for the somewhat contrasting findings is the lower statistical power in the age-stratified analyses. The wider 95% CIs in these analyses indicate that the age-stratified results should be interpreted with some caution.

Numerous studies have found pain in different body regions, e.g. low-back, neck/shoulders, and knees, to be debilitating among various job groups including healthcare workers [8–10]. Both longer duration [20] and higher intensity [9] of pain in the low-back, neck/shoulders, and knees increases the risk of long-term sickness absence, where a dose-response association was observed for all body regions [9]. Furthermore, both single- and multisite pain predict future disability pension in different job groups, with higher number of pain regions leading to increased risk of disability pension [15, 16, 40]. The present study investigated specific pain regions that were mutually adjusted and found strong associations to disability pension in workers with pain in all investigated body regions. Including that higher pain intensity in low-back, neck/shoulders, and legs associate with lower work ability [28, 29, 31], all these findings underscore the importance of targeted focus on reducing the pain prevalence and more specifically pain *intensity* among eldercare workers to retain good health and hence prolong the working life. Furthermore, because of the high prevalence of pain and disability pension among eldercare workers, eldercare workers should be considered in suitable pension reforms if they are not capable of working until the statutory pension age.

The present study’s findings that musculoskeletal pain *intensity* in different body regions function as a predictor for future disability pension among eldercare workers supplements previous findings that high physical work demands [21, 22], pain duration [23], and multi-site pain [15] predict disability pension. As a novel finding, the present study found high/very high pain intensity to predict disability pension among both younger and older workers. Additionally, higher physical work demands and musculoskeletal pain intensity in the low-back [28], neck/shoulders [31], and legs [29] associated with lower work ability compared to workers with the same pain intensity but less physically demanding work, i.e. workers with physically demanding occupations are more affected by their pain. Since eldercare work is physically demanding, and work ability function as a proxy for disability pension, our findings elaborate on these findings that pain intensity may have a debilitating effect on work ability and consequently leads to early retirement for workers with physically demanding occupations. The sample of the present study included only those directly involved in care work, and the sample is large and spread across the country. Thus, it is reasonable to assume that the sample is representative for eldercare workers in Denmark. ‘Human health and social work activities’ in Denmark is fairly close to the average EU-27 for many work-related factors [41]. Thus, the generalizability of the present findings may also, although with certain caution, be extended to the European countries as a whole. However, the present study do not have information on the diagnosis for disability pension, meaning that we cannot conclude that the disability pensions are due to musculoskeletal pain. The study show that more intense musculoskeletal pain is associated with *higher risk* of disability pension within 11 years of follow-up.

The PAR analyses indicate a potential in saving people from leaving the labour market prematurely. Workplace- and societal initiatives could prolong the working life for many workers and should be prioritized to improve the organisation and physical work environment of eldercare work. For example, the use of technical assistive devices for patients transfer is associated with reduced physical workload and significantly decreases the risk for back injury and low-back pain [42, 43], whereas a lack of assistive devices and ability to use the devices is a common contributing factor for back injury events among eldercare workers [44, 45]. Another relevant initiative to prevent and reduce pain is physical exercise at the workplace, particularly strength training, which also improves the physical capacity through increased muscle strength and muscular endurance among workers with physically demanding occupations [46, 47], whereby the work tasks, e.g. lifting and assisting people, are performed at a lower relative strain. In fact, low physical capacity function as an increased risk factor for future disability pension [48]. Thus, a higher physical capacity through physical exercise could potentially prolong the working life since workers with less physically demanding occupations retire later than workers with hard physical work [4]. Furthermore, an improved physical capacity will benefit the workers during the inherent age-related loss in muscle mass and strength with aging (sarcopenia) [49–51] and postpone the point when reaching the “disability threshold” where the workers are unable to meet their physical work demands [49, 50]. Another initiative to reduce the pain intensity among eldercare workers, and thereby potentially prolong their working life, could be to adjust the work tasks to the worker’s physical capacity. Adjusting the work tasks would reduce the physical strain, which may increase the work ability and reduce the risk of leaving the labour market temporarily or permanently. Lastly, reallocating eldercare workers with a high level of musculoskeletal pain to other job functions with less physical demands may also be a suitable solution to lower the pain and reduce the risk of disability pension.

### Limitations and strengths

The present study contains some methodological limitations and strengths. The homogenous group of female eldercare workers in eldercare implies that the findings only apply to the included population of female eldercare workers and cannot be generalized to the general working population. However, the inclusion of this homogenous group reduces the risk of socioeconomic confounding. A further limitation of the study may be that our data only pertains to currently employed eldercare workers and do not contain information on lifetime work exposures (preceding or succeeding eldercare work) that potentially may influence the risk of disability pension. Moreover, the present results may be caused by other underlying factors such as poor health or chronic diseases. However, excluding those with poor health and chronic conditions at baseline also leads to excluding many workers with pain. Therefore, such an approach would create a selected group of completely “healthy” respondents where some would still report high pain of unknown cause, i.e. maybe not being completely healthy (unidentified chronic disease). Thus, as the cause of pain in many cases is unknown, we chose to include all workers – in spite of known or unknown chronic disease - and use pain intensity as the predictor variable. A strength of the study is the use of the high-quality DREAM to register disability pension during an 11-year period. In addition, investigating the risk of disability pension from pain intensity in various body regions (low-back, neck/shoulders, and knees) strengthens the study and increases the specificity of the study in order to improve the physical working environment among eldercare workers. At last, the calculation of PAR is a strength as it increases our understanding of the consequences of work-related musculoskeletal pain.

## Conclusions

The present study demonstrates a positive dose-response association between pain intensity in low-back, neck/shoulders, and knees, and risk of disability pension among female eldercare workers. Reducing pain levels among eldercare workers can reduce the risk of future disability pension and prevent that a substantial proportion of eldercare workers are pushed out of the labour market prematurely. Improving the physical working environment, reorganising work tasks, and promoting a healthy lifestyle can help to reduce high prevalence and intensity of pain among eldercare workers, and ultimately ensure a long and healthy working life.

## Data Availability

Data are available upon reasonable request. The authors encourage collaboration and use of the data by other researchers. Data are stored on the server of Statistics Denmark, and researchers interested in using the data for scientific purposes should contact the project leader Prof. Lars L. Andersen, lla@nfa.dk.

## Abbreviations

HR: Hazard ratio
CI: Confidence interval
SD: Standard deviation
DREAM: Danish Register for Evaluation of Marginalization
BMI: Body mass index
PAR: Population attributable risk
NRS: Numeric rating scale
